# Supplementary material for: Factors associated with hypotension during the first hour of continuous renal replacement therapy in critically Ill patients: A prospective observational study
Source: PLoS One. 2025 Jun 26;20(6):e0324235. doi: 10.1371/journal.pone.0324235 (PMC12200669; doi:10.1371/journal.pone.0324235)
Supplement: S1 Table — (DOCX) [file pone.0324235.s001.docx]

### Supplementary

**S1 Table. The coefficients of LASSO regression analysis**

| Characteristics | Coefficient |
| --- | --- |
| (Intercept) | -0.77359113 |
| Age | 0.25408053 |
| Gender (female) | -0.55458616 |
| Duration of ICU stay at CRRT initiation | 0.02978852 |
| APACHE Ⅱ on the day of ICU admission | 0.15646045 |
| APACHE Ⅱ on the day of CRRT initiation | 0 |
| SOFA on the day of CRRT initiation | 0.07407726 |
| Mechanical ventilation (yes) | 0.54200532 |
| Use of colloidal solution (yes) | -1.18692234 |
| Use of vasopressors (yes) | 0.339472327 |
| vasoactive inotropic score | 0 |
| Ultrafiltration rate (101-200 ml/h) | -0.51883188 |
| Ultrafiltration rate (201-300 ml/h) | -0.24924369 |
| Ultrafiltration rate (>300 ml/h) | -1.74700163 |
| pH | 0.01649326 |
| White blood cell | 0.01258652 |
| Hemoglobin | -0.1514889 |
| Platelet | -0.3344077 |
| C-reactive protein | 0.06095290 |
| Phosphate | 0 |
| International normalized ratio | 0.15893124 |
| Myoglobin | 0.26932355 |
| Systolic blood pressure | -0.41531003 |
| Diastolic blood pressure | -0.52736532 |
| Mean arterial pressure | 0 |
| Respiratory rate | 0.270689191 |
