# Supplementary material for: Factors associated with hypotension during the first hour of continuous renal replacement therapy in critically Ill patients: A prospective observational study
Source: PLoS One. 2025 Jun 26;20(6):e0324235. doi: 10.1371/journal.pone.0324235 (PMC12200669; doi:10.1371/journal.pone.0324235)
Supplement: Attachment — Submitted filename: PONE-D-25-05284 edit.pdf [file pone.0324235.s002.pdf]

PLOS ONE

Factors Associated with Hypotension During the Initial Hour of Continuous Renal Replacement Therapy in Critically Ill Patients: A Prospective Observational Study

--Manuscript Draft--

**Manuscript Number:**	PONE-D-25-05284
**Article Type:**	Research Article
**Full Title:**	Factors Associated with Hypotension During the Initial Hour of Continuous Renal Replacement Therapy in Critically Ill Patients: A Prospective Observational Study
**Short Title:**	Factors Associated with Hypotension During the Initial Hour of CRRT in Critically Ill Patients
**Corresponding Author:**	Jingjuan Xu<br>Third Affiliated Hospital of Soochow University: Changzhou First People's Hospital<br>Changzhou, Jiangsu CHINA
**Keywords:**	Continuous renal replacement therapy; Hypotension; Risk factors; Critical care; Lasso regression
**Abstract:**	Purpose<br>To investigate the factors of hemodynamic instability within the initial hour of continuous renal replacement therapy (CRRT) in critically ill patients.<br>Materials and methods<br>A prospective observational cohort study of patients admitted to the intensive care unit (ICU) and underwent CRRT between January 17, 2024, and December 25, 2024, was conducted. The least absolute shrinkage and selection operator (LASSO) regression was used to screen potential factors and a multivariate logistic regression model was performed to determine the independent factors of hypotension within the initial hour of CRRT.<br>Results<br>Hypotension occurred in 166 out of 435 patients (38.2%). Female gender (OR=0.53, 95%CI:0.3 ~ 0.89), the use of colloidal solution (OR=0.23, 95%CI:0.12 ~ 0.46), platelet count (PLT) (OR=0.99, 95%CI:0.99 ~ 0.99), and baseline mean arterial pressure (MAP) (OR=0.95, 95%CI:0.93 ~ 0.96) were recognized as protective factors against hypotension within the initial hour of CRRT in critically ill patients. Older age (OR=1.02, 95%CI:1.01 ~ 1.04), mechanical ventilation (OR=2.59, 95%CI:1.28 ~ 5.23), ultrafiltration rates of 101-200 mL/h (OR=2.04, 95%CI:1.11 ~ 3.75), international normalized ratio (INR) (OR=1.78, 95%CI:1.02 ~ 3.09), and high myoglobin level (OR=1.01, 95%CI:1.01 ~ 1.01) were identified as significant risk factors. Baseline MAP and mechanical ventilation are the most important predictors of hypotension within the initial hour of CRRT in critically ill patients.<br>Conclusions<br>The incidence of hypotension within the initial hour of CRRT in critically ill patients was 38.2%. Female gender, PLT, baseline MAP are protective factors, and age, mechanical ventilation, ultrafiltration rate of 101-200ml/h, INR, myoglobin are risk factors for hypotension within the initial hour of CRRT in critically ill patients.
**Order of Authors:**	Jingjuan Xu
	Mengdie Xue
	Suying Lu
	Chenglin Zhao
	Zheyao Zhang
	Jing Yang
	Zhiyu Mao
**Additional Information:**	
**Question**	**Response**

Powered by Editorial Manager® and ProduXion Manager® from Aries Systems Corporation

**Financial Disclosure**<br><br>Enter a financial disclosure statement that describes the sources of funding for the work included in this submission. Review the submission guidelines for detailed requirements. View published research articles from *PLOS ONE* for specific examples.<br><br>This statement is required for submission and **will appear in the published article** if the submission is accepted. Please make sure it is accurate.<br><br>**Funded studies**<br>Enter a statement with the following details:<br>• Initials of the authors who received each award<br>• Grant numbers awarded to each author<br>• The full name of each funder<br>• URL of each funder website<br>• Did the sponsors or funders play any role in the study design, data collection and analysis, decision to publish, or preparation of the manuscript?<br><br>Did you receive funding for this work?	The author(s) received no specific funding for this work.
**Competing Interests**<br><br>Use the instructions below to enter a competing interest statement for this submission. On behalf of all authors, disclose any competing interests that could be perceived to bias this work—acknowledging all financial support and any other relevant financial or non-financial competing interests.<br><br>This statement is required for submission and **will appear in the published article** if the submission is accepted. Please make sure it is accurate and that any funding sources listed in your Funding Information later in the submission form are also declared in your Financial Disclosure statement.	The authors have declared that no competing interets exist.

View published research articles from *PLOS ONE* for specific examples.<br><br>**NO authors have competing interests**<br><br>Enter: *The authors have declared that no competing interests exist.*<br><br>**Authors with competing interests**<br><br>Enter competing interest details beginning with this statement:<br><br>*I have read the journal's policy and the authors of this manuscript have the following competing interests: [insert competing interests here]*<br><br>* typeset	
**Ethics Statement**<br><br>Enter an ethics statement for this submission. This statement is required if the study involved:<br><br>• Human participants<br>• Human specimens or tissue<br>• Vertebrate animals or cephalopods<br>• Vertebrate embryos or tissues<br>• Field research<br><br>Write "N/A" if the submission does not require an ethics statement.<br><br>General guidance is provided below. Consult the submission guidelines for detailed instructions. **Make sure that all information entered here is included in the Methods section of the manuscript.**	This study was approved by the Ethics Committee of the Third Affiliated Hospital of Soochow University (Approval No.: F-IRB-SOP-00710). Written informed consent was obtained from all participants or their legal representatives. Data were anonymized and analyzed in compliance with institutional guidelines and the Declaration of Helsinki.

Powered by Editorial Manager® and ProduXion Manager® from Aries Systems Corporation

**Format for specific study types**<br><br>**Human Subject Research (involving human participants and/or tissue)**<br>• Give the name of the institutional review board or ethics committee that approved the study<br>• Include the approval number and/or a statement indicating approval of this research<br>• Indicate the form of consent obtained (written/oral) or the reason that consent was not obtained (e.g. the data were analyzed anonymously)<br><br>**Animal Research (involving vertebrate animals, embryos or tissues)**<br>• Provide the name of the Institutional Animal Care and Use Committee (IACUC) or other relevant ethics board that reviewed the study protocol, and indicate whether they approved this research or granted a formal waiver of ethical approval<br>• Include an approval number if one was obtained<br>• If the study involved *non-human primates*, add *additional details* about animal welfare and steps taken to ameliorate suffering<br>• If anesthesia, euthanasia, or any kind of animal sacrifice is part of the study, include briefly which substances and/or methods were applied<br><br>**Field Research**<br><br>Include the following details if this study involves the collection of plant, animal, or other materials from a natural setting:<br>• Field permit number<br>• Name of the institution or relevant body that granted permission	
**Data Availability**<br><br>Authors are required to make all data underlying the findings described fully available, without restriction, and from the time of publication. PLOS allows rare exceptions to address legal and ethical concerns. See the PLOS Data Policy and FAQ for detailed information.	No - some restrictions will apply

A Data Availability Statement describing where the data can be found is required at submission. Your answers to this question constitute the Data Availability Statement and **will be published in the article**, if accepted.<br><br>**Important:** Stating 'data available on request from the author' is not sufficient. If your data are only available upon request, select 'No' for the first question and explain your exceptional situation in the text box.<br><br>Do the authors confirm that all data underlying the findings described in their manuscript are fully available without restriction?	
**Describe where the data may be found in full sentences. If you are copying our sample text, replace any instances of XXX with the appropriate details.**<br><br>• If the data are **held or will be held in a public repository**, include URLs, accession numbers or DOIs. If this information will only be available after acceptance, indicate this by ticking the box below. For example: *All XXX files are available from the XXX database (accession number(s) XXX, XXX.*).<br>• If the data are all contained **within the manuscript and/or Supporting Information files**, enter the following: *All relevant data are within the manuscript and its Supporting Information files.*<br>• If neither of these applies but you are able to provide **details of access elsewhere**, with or without limitations, please do so. For example:<br><br>*Data cannot be shared publicly because of [XXX]. Data are available from the XXX Institutional Data Access / Ethics Committee (contact via XXX) for researchers who meet the criteria for access to confidential data.*<br><br>*The data underlying the results presented in the study are available from (include the name of the third party*	The datasets are not publicly available due to the potential for loss of privacy as required by the research ethics committee. The data are available from the corresponding author and research ethics committee (helenclinical@163.com) on reasonable request.

and contact information or URL).<br>• This text is appropriate if the data are owned by a third party and authors do not have permission to share the data.<br><br>* typeset	
Additional data availability information:	

Manuscript Click here to access/download;Manuscript;Main text.docx

Factors Associated with Hypotension During the Initial Hour of Continuous Renal Replacement Therapy in Critically Ill Patients: A Prospective Observational Study

Mengdie Xue[1]; Suying Lu[2]; Chenglin Zhao[2]; Zheyao Zhang[3]; Jing Yang[2]; Zhiyu Mao[1]; Jingjuan XU[2]

[1] School of Nursing, Suzhou Medical College of Soochow University, Suzhou, China

[2] The Third Affiliated Hospital of Soochow University, Changzhou, China

[3] School of Medical Imaging, Xuzhou Medical University, Xuzhou, China

Correspondence

Jingjuan Xu, The Third Affiliated Hospital of Soochow University, Changzhou, China

E-mail: heleneshue@sina.com

Abstract

Purpose

To investigate the factors of hemodynamic instability within the initial hour of continuous renal replacement therapy (CRRT) in critically ill patients.

Materials and methods

A prospective observational cohort study of patients admitted to the intensive care unit (ICU) and underwent CRRT between January 17, 2024, and December 25, 2024, was conducted. The least absolute shrinkage and selection operator (LASSO) regression was used to screen potential factors and a multivariate logistic regression model was performed to determine the independent factors of hypotension within the initial hour of CRRT.

Results

Hypotension occurred in 166 out of 435 patients (38.2%). Female gender (*OR*=0.53, 95%*CI*:0.3 ~ 0.89), the use of colloidal solution (*OR*=0.23, 95%*CI*:0.12 ~ 0.46), platelet count (PLT) (*OR*=0.99, 95%*CI*:0.99 ~ 0.99), and baseline mean arterial pressure (MAP) (*OR*=0.95, 95%CI:0.93 ~ 0.96) were recognized as protective factors against hypotension within the initial hour of CRRT in critically ill patients. Older age (*OR*=1.02, 95%*CI*:1.01 ~ 1.04), mechanical ventilation (*OR*=2.59, 95%*CI*:1.28 ~ 5.23), ultrafiltration rates of 101-200 mL/h (*OR*=2.04, 95%*CI*:1.11 ~ 3.75), international normalized ratio (INR) (*OR*=1.78, 95%*CI*:1.02 ~ 3.09), and high myoglobin level (*OR*=1.01, 95%*CI*:1.01 ~ 1.01) were identified as significant risk factors. Baseline

MAP and mechanical ventilation are the most important predictors of hypotension within the initial hour of CRRT in critically ill patients.

Conclusions

The incidence of hypotension within the initial hour of CRRT in critically ill patients was 38.2%. Female gender, PLT, baseline MAP are protective factors, and age, mechanical ventilation, ultrafiltration rate of 101-200ml/h, INR, myoglobin are risk factors for hypotension within the initial hour of CRRT in critically ill patients.

Keywords

Continuous renal replacement therapy; Hypotension; Risk factors; Critical care; Lasso regression

Introduction

Continuous Renal Replacement Therapy (CRRT) is a group of therapies that continuously and gradually remove solutes and excess fluids from the body[1]. It is a crucial therapeutic intervention for multiorgan function support and serves as the principal modality of Renal Replacement Therapy (RRT) for critically ill patients[2, 3]. Compared to other dialysis modalities, CRRT exhibits superior hemodynamic stability[4, 5]. Nonetheless, the incidence of intra-dialytic hypotension (IDH) during CRRT remains high, varying between 43% to 87%[6]. The presence of IDH influences the adequacy and efficacy of dialysis[7], and exacerbates cardiovascular complications and organ dysfunction[8, 9], which impacts the quality of life and prognosis for critically ill patients. The incidence of IDH is higher within the initial hour of CRRT.

Studies have shown that almost two-thirds of patients experience IDH within the initial hour of CRRT, which is independently linked to heightened in-hospital mortality[10-12].

Research on IDH during CRRT is still constrained, predominantly comprising retrospective studies. These studies are often subject to information and selection biases, and their findings may differ according to diagnostic criteria for IDH[6, 10, 13-15]. The occurrence of IDH in critically ill patients is affected by a combination of factors, and previous studies have not accounted for the covariance among those factors. This study analyzed the incidence of IDH in critically ill patients within the initial hour of CRRT. Least absolute shrinkage and selection operator (LASSO) regression was used to screen the potential influencing factors and reduce collinearity, and then explored the independent variables associated with hypotension. This study was expected to provide valuable information to mitigate the occurrence of IDH during the commencement of CRRT.

Methods

Study design and population

This prospective observational study involved a cohort of adult patients (≥18 years old) undergoing CRRT, admitted to the central intensive care unit (ICU) of the Third Affiliated Hospital of Soochow University from January 17, 2024, to December 25, 2024. The Institutional Ethics Committee approved the project (No. F-IRB-SOP-00710), and all patients or their relatives signed the informed consent form.

Inclusion and exclusion criteria

The inclusion criteria for the study were as follows:(1) patients aged ≥18 years old; (2) APACHE II score ≥15 at the day of ICU admission; (3) arterial cannulation for real-time hemodynamic monitoring;

The study's exclusion criteria included: (1) central nervous system disorders; (2) the use of extracorporeal membrane oxygenation (ECMO), ventricular assist devices, or pacemakers; and (3) hypotensive (mean arterial pressure <65 mmHg) when starting CRRT.

The study's dropout criteria included: (1) mortality during the observation period (the initial hour of CRRT); (2) cessation of CRRT for non-hemodynamic reasons; (3) alteration in ventilation or sedation during the observation period; and (4) premature intervention before meeting diagnostic criteria for hypotension.

Definition of hypotension within the initial hour of CRRT

There is no definitive definition of IDH. The prevalent definition of IDH, as articulated by the Kidney Disease Outcomes Quality Initiative (KDOQI) of the National Kidney Foundation (USA), is characterized by a decrease in systolic blood pressure (SBP) of ≥20 mmHg or mean arterial pressure (MAP) of ≥10 mmHg, accompanied by symptoms such as abdominal discomfort, yawning, sighing, nausea, vomiting, muscle cramps, restlessness, dizziness, and anxiety necessitating intervention[16]. This definition is inapplicable to critically ill patients, as they cannot articulate pertinent symptoms due to impaired mental status or mechanical ventilation. Moreover, significant

discrepancies exist in SBP measured at various arteria cannulation sites, while MAP exhibits lesser variations[17]. It has been demonstrated that a MAP below 65 mmHg serves as the threshold for the onset of new acute myocardial and renal injury[18]. Thus, we defined IDH occurring within the initial hour of CRRT as a new MAP<65 mmHg that endures for over one minute, consistent with the definition by Chazot et al.[15].

Experimental methods

Instruments and Equipment

The instruments and equipment used in this study included: (1) Hemodialysis machine and tubing: Gambro Hemodialysis Machine Prismaflex with corresponding tubing (2) Filters: Gambro M100 or M150; (3) Monitors: Mindray Benevision N15; (4) Arterial pressure transducer: icumedical,01C-42584-25

Procedure for CRRT operation

The CRRT operation adhered to the Continuous Renal Replacement Therapy Nursing Group Standard established by the Chinese Nursing Association in 2023[19]. Relevant operations were performed by nurses qualified for CRRT. The parameters were set according to medical advice.

Data collection

We developed the data collection form by reviewing pertinent literature on the determinants of CRRT-associated hypotension in databases including China National

Knowledge Infrastructure (CNKI), Wanfang, and PubMed et al., combined with clinical practice and discussion during the expert meeting, to identify the potential influencing factors for hypotension in critically ill patients undergoing CRRT[6, 10-13, 15, 20, 21]. The form encompassed (1) clinical characteristics: gender, age, body mass index (BMI), Acute Physiology, and Chronic Health Evaluation II (APACHE II) at ICU admission day, duration of ICU stay at CRRT initiation, age-adjusted Charlson Comorbidity Index (aCCI), comorbidities (hypertension, diabetes, heart failure), and APACHE II and Sequential Organ Failure Assessment (SOFA) on the day of CRRT; (2) treatments and interventions: use of vasopressors, vasoactive inotropic score (VIS), use of colloid solution, and mechanical ventilation. (3) laboratory results: lactate, PH, $PaCO_2$, white blood cell count (WBC), platelet count (PLT), hemoglobin (Hb), albumin, C-reactive protein (CRP), blood urea nitrogen (BUN), serum natrium (Na), serum kalium (K), serum calcium (Ca), serum phosphate (P), N-terminal pro-B-type natriuretic peptide (NT-proBNP), international normalized ratio (INR), myoglobin, and troponin I; (4) CRRT parameters: CRRT modality, initial blood flow rate, therapeutic blood flow rate, replacement fluid rate, ultrafiltration rate;(5) baseline vital signs: temperature, heart rate (HR), SBP, diastolic blood pressure (DBP), MAP, and respiratory rate (RR).

Prior to data collection, researchers underwent homogenization training to achieve consistency and standardization in the data collection process. Clinical characteristics and laboratory results were extracted from the hospital's medical records system. Laboratory results were chosen from the latest tests conducted within 24 hours before

the start of CRRT. Treatments and interventions were assessed and recorded before the CRRT initiation; CRRT parameters were recorded following parameter adjustments. Before initiating CRRT, patients were maintained in a tranquil state and positioned in a semi-recumbent posture. Subsequently, arterial blood pressure was calibrated and set to zero. Baseline vital signs were documented when the waveform exhibited stability. Vital signs were continuously observed by a designated researcher until the occurrence of IDH or the end of the observation period. Non-urgent operations were mostly circumvented to reduce interference. All data were examined and documented by two researchers. The IDH and non-IDH groups were categorized based on the occurrence of IDH throughout the observation period.

Statistical analysis

Missing data were imputed using multiple interpolation methods. Continuous variables were summarized as mean and standard deviation (mean ± SD) or median and interquartile range [M (Q_1, Q_3)], contingent upon the normality of the data distribution, while categorical variables were reported as counts and percentages [n(%)]. The independent *t*-test or Wilcoxon rank-sum test was used for continuous variables, and the Chi-squared test or *Fisher's* exact test was applied for categorical variables.

Variables exhibiting statistically significant variations between the two groups ($P<0.05$) were subsequently analyzed using LASSO regression to ascertain probable important factors. LASSO regression systematically identifies a subset of variables by applying an L1-norm penalty, hence mitigating any collinearity. The tuning parameter lambda

(λ), which dictates the extent of shrinkage, was chosen by 10-fold cross-validation. Variables were preserved according to the coefficients associated with the minimum lambda (λ).

Multivariate logistic regression with stepwise backward selection was conducted to identify independent risk factors for IDH during the initial hour of CRRT. We used Shapley Additive Explanations (SHAP) values to illustrate the importance of each variable in the model. A two-sided *P*-value <0.05 was considered statistically significant. All statistical analyses were performed using *R* Studio.

Results

A total of 496 patients of CRRT were recorded in this study, with 31 excluded and 30 dropped, leaving 435 for the final analysis **(Figure 1)**. Among these, 166 patients developed IDH, representing a prevalence rate of 38.2%. The median age of patients was 71 (58,78), and 308 patients (70.64%) were male. The median APACHE II score on the day of ICU admission was 28 (21,33). There were 300 (68.97%) patients with hypertension and 180 (41.28%) with diabetes. Detailed characteristics are presented in **Table 1.**

Table 1 The characteristics of patients (n=436)

Characteristics	Values
Age, years [M (Q_1, Q_3)]	71 (58, 78)
Male [n (%)]	308 (70.64)
BMI, kg/m^2 [Mean ± SD]	23.60 ± 4.02
APACHE II at ICU admission day [M (Q_1, Q_3)]	28 (21, 33)
Duration of ICU stay at CRRT initiation, day [M (Q_1, Q_3)]	4 (1, 9)
Mechanical ventilation [n (%)]	32 (73.79)
aCCI [Mean ± SD]	5.06 ± 2.92
Comorbidities	
Hypertension [n (%)]	300 (68.97)
Diabetes [n (%)]	180 (41.28)
Heart failure [n (%)]	170 (38.99)

* BMI, body mass index; APACHE, acute physiology, and chronic health evaluation; ICU, intensive care unit; CRRT, Continuous Renal Replacement Therapy; aCCI, age-adjusted Charlson Comorbidity Index

Comparison between the two groups revealed that patients in the IDH group had more severe conditions and prolonged ICU stays before CRRT initiation than the non-IDH group. Additionally, the IDH group exhibited lower levels of Hb, PLT, and baseline blood pressure, along with higher levels of CRP and myoglobin. The proportions of males (79.52% vs 65.06%, p=0.001), the use of vasopressors (78.92% vs 64.68%, p=0.002), mechanical ventilation (80.12% vs 69.89%, p=0.018) were significantly

higher in the IDH group. In contrast, the proportion of the use of colloidal solution was lower in the IDH group. The details are presented in **S1 Table.**

We performed LASSO regression analysis using the 21 factors that showed significant differences between the two groups, including age, gender, APACHE II at ICU admission day, duration of ICU stay at CRRT initiation, APACHE II and SOFA on the day of CRRT initiation, VIS, the use of vasopressors, the use of colloidal solution, mechanical ventilation, Hb, PLT, CRP, P, INR, myoglobin, ultrafiltration rate, RR, SBP, DBP, and MAP. **Figure 2A** displays the cross-validation error, and **Figure 2B** illustrates the coefficient trajectories in the LASSO regression model. The coefficients are presented in **S2 Table**. The results indicated that, when the tuning parameter λ was set to 0.036 (one standard error of the minimum λ), 19 variables were selected in the model, except for APACHE II on the day of CRRT initiation and baseline MAP. Although baseline MAP was excluded, we believe that SBP and DBP have a strong correlation. Given the definition of hypotension in this study, we propose that baseline MAP may serve as a more reliable predictor. Therefore, we decided to exclude SBP and DBP and include MAP in the model.

We compared the performance of models including either SBP or MAP. The model including MAP showed a lower Akaike Information Criterion (AIC) of 466.76, which is better than the model with SBP (AIC: 472.52). The result of the multivariate logistic regression model with MAP included is shown in **Table 2**. Female (*OR*=0.53, 95%*CI*:0.3~0.89), the use of colloidal solution (*OR*=0.23, 95%*CI*:0.12 ~ 0.46), PLT (*OR*=0.99, 95%*CI*:0.99 ~ 0.99), and baseline MAP (*OR*=0.95, 95%*CI*:0.93 ~ 0.96) were

recognized as protective factors against IDH. Older age (*OR*=1.02, 95%*CI*:1.01 ~ 1.04), mechanical ventilation (*OR*=2.59, 95%*CI*:1.28 ~ 5.23), ultrafiltration rates of 101-200 mL/h (*OR*=2.04, 95%*CI*:1.11 ~ 3.75), INR (*OR*=1.78, 95%*CI*:1.02 ~ 3.09), and myoglobin levels (*OR*=1.01, 95%*CI*:1.01 ~ 1.01) were identified as significant risk factors for IDH.

Table 2 Multivariate Logistic Regression Analysis of IDH

**Variables**	***β***	***S. E***	***Z***	***P***	***OR* (95%*CI*)**
Intercept	-0.98	1.39	-0.71	0.478	0.37 (0.02~5.66)
Age, year	0.02	0.01	2.73	**0.006**	1.02 (1.01 ~ 1.04)
Gender					
male					1.00 (Reference)
female	-0.65	0.28	-2.36	**0.018**	0.53 (0.3 ~ 0.89)
Mechanical ventilation					
no					1.00 (Reference)
yes	0.95	0.35	2.68	**0.007**	2.59 (1.28 ~ 5.23)
Use of Colloidal solution					
no					1.00 (Reference)
yes	-1.45	0.36	-4.06	**<0.001**	0.23 (0.12 ~ 0.46)
Use of vasopressors					
no					1.00 (Reference)
yes	0.49	0.33	1.48	0.138	1.64 (0.85 ~ 3.14)
Ultrafiltration rate, ml/h					

0-100					1.00 (Reference)
101-200	0.71	0.31	2.30	**0.022**	2.04 (1.11 ~ 3.75)
201-300	0.10	0.53	0.19	0.847	1.11 (0.39 ~ 3.1)
>300	-14.56	860.82	-0.02	0.987	0.00 (0.00 ~ Inf)
Hb, g/L	-0.01	0.01	-1.74	0.082	0.99 (0.98 ~ 1.00)
PLT, 10^9/L	-0.01	0.00	-3.14	**0.002**	0.99 (0.99 ~ 0.99)
INR	0.58	0.28	2.04	**0.041**	1.78 (1.02 ~ 3.09)
Myoglobin, ng/ml	0.01	0.00	3.07	**0.002**	1.01 (1.01 ~ 1.01)
MAP, mmHg	-0.05	0.01	-5.93	**<0.001**	0.95 (0.93 ~ 0.96)
RR, rpm	0.09	0.03	2.95	**0.003**	1.10 (1.03 ~ 1.16)

**S. E*, standard error; *OR*, odds ratio; *CI*, confidence interval; Hb, hemoglobin; PLT, platelet count; INR, international normalized ratio; MAP, mean arterial pressure; RR, respiratory rate.

SHAP values were computed to evaluate the importance of each predictor in the model. A bar plot **(Figure 3)** was generated to visualize the importance of the predictors, with features ranked according to their average impact on the model's output. The results indicate that baseline MAP and mechanical ventilation are the two most important predictors.

Discussion

CRRT is widely used in critically ill patients owing to its superior hemodynamic stability compared to other dialysis modalities. During CRRT, patients typically lose approximately 150 to 200 mL of circulating blood volume[22]. This rapid decrease in blood volume may not be compensated for critically ill patients, which causes hemodynamic instability. Hypotension can occur even with interventions such as bidirectional blood withdrawal. There is no standardized definition of hypotension during CRRT in critically ill patients. Depending on different definitions, previous studies have reported the incidence of hypotension within the initial hour of CRRT ranging from 8.4% to 64.6%[10-12, 23]. This study found that the incidence of hypotension within the initial hour of CRRT in critically ill patients was 38.2%. Identifying factors of IDH within the initial hour of CRRT and meticulously observing hemodynamic changes at the start of CRRT are essential for implementing prompt and effective preventive strategies.

The results of this study showed that gender and age are independent influencing factors for hypotension during the initial hour of CRRT in critically ill patients. In this study, female gender was considered as a protective factor (*OR*:0.53, 95%*CI*: 0.3–0.89), which is consistent with the findings of Juan C et.al (*OR*:0.43, 95%*CI*: 0.3–0.89) [24].In addition, our findings align with previous studies[6, 11, 20, 25, 26], which demonstrated an independent correlation between age and the occurrence of hypotension during CRRT. Jing Li et al. reported that patients aged ≥60 years old have a higher risk of hypotension during CRRT (*OR*: 6.125, 95%*CI*: 2.51–14.97) [6]. This is primarily

associated with the physiological decline observed in the elderly, characterized by changes in myocardial function, including diminished myocardial contractility and left ventricular diastolic dysfunction. Simultaneously, diminished arterial endothelial function leading to decreased vascular compliance, along with autonomic dysfunction, impairs the rapid compensatory mechanisms for hemodynamic alterations in elderly patients, significantly reducing the ability to endure fluctuations in blood volume [27]. In addition, elderly patients frequently present with a variety of chronic diseases, which further increases the risk of hypotension during CRRT[28].

Mechanical ventilation was recognized as an independent risk factor for hypotension during the initial hour of CRRT in critically ill patients, aligning with the findings of Kelly et al[13]. In critically ill patients, mechanical ventilation implies severe respiratory failure. According to the mechanism of cardiopulmonary interaction, mechanical ventilation can increase intrathoracic negative pressure and decrease return blood volume, which may lead to severe hemodynamic consequences in certain clinical settings, such as chronic obstructive pulmonary disease (COPD), acute respiratory distress syndrome (ARDS), and acute left heart failure[29]. This study also found that the use of colloidal solution (e.g., albumin, low molecular weight dextran, etc.) reduced the incidence of hypotension, corroborating earlier studies [6, 11, 20, 23]. Due to higher molecular weight and stronger colloidal osmotic pressure, colloidal solution are effective in sustaining intravascular volume, and reducing the occurrence of hypotension[30]. For patients with circulatory failure, selecting the suitable colloidal

solution based on the specific clinical situation before CRRT initiation may help reduce the risk of hypotension.

This study found PLT as a protective factor. Besides its function in hemostasis and coagulation, PLT plays an important role in preserving the integrity of the vascular endothelium by releasing vascular endothelial growth factor (VEGF). The VEGF generated by PLT facilitates endothelial cell repair and proliferation, hence reduce minimizing damage and leaking of the vessel wall[31]. This helps to mitigate the hemodynamic instability caused by fluid removal during CRRT. Kang et al. found INR as a risk factor for hypotension in their machine learning model[23], aligning with this study's findings, although it was ranked lower in terms of importance. Myoglobin was identified for the first time as a risk factor for hypotension within the initial hour of CRRT in critically ill patients in this study. This may be related to the higher proportion of infections in critically ill patients and the impairment of cardiomyocytes by inflammatory mediators. A global epidemiological analysis of ICU infection indicated that around 54% of ICU patients had suspected or confirmed infections[32]. Myoglobin not only has a direct toxic effect on the kidney, but its damage to endothelial cells and microvessels exacerbates hemodynamic instability.

Several studies have confirmed that ultrafiltration rate is an independent risk factor for hypotension during CRRT in critically ill patients[6, 10, 11, 20], with rates ≥100 ml/h significantly increasing the risk of hypotension[6, 20]. The results of this study show that the risk of hypotension was significantly increased when the ultrafiltration rate was 101-200 ml/h compared to the ultrafiltration rate of 0-100 ml/h. However, the

ultrafiltration rate of >200 ml/h did not show any significant differences, which may be due to the insufficient sample size. High ultrafiltration rates imply that more fluid is removed, and critically ill patients may not be able to compensate for the rapid decline in blood volume. Sun et al. proposed a three-stage volume management strategy that provides important guidance for individualized ultrafiltration, suggesting that the net ultrafiltration rate for CRRT should start at a low dose, with an initial net ultrafiltration rate of 80 (49,111) ml/h for hemodynamically unstable patients[33], to reduce the risk of hypotension and provide a more balanced and individualized ultrafiltration plan for patients.

In this study, the use of vasopressors was not found to be an independent risk factor for hypotension during the first hour of CRRT, a variable that may have a strong correlation with baseline blood pressure (MAP). It still had a significant impact on the occurrence of hypotension. Previous studies have shown that patients receiving vasopressors were at higher risk of hypotension[6, 10, 11]. One-third of ICU patients experience circulatory failure and require vasopressors for main organ perfusion[34], which may mask the true hemodynamic instability of the patient. The filter's operation can affect the pharmacokinetics of vasopressors. Vasopressors with high unbound fractions (e.g., epinephrine and norepinephrine) are more likely to be effectively removed by CRRT[35]. The present feature ranking analysis demonstrated that baseline MAP is the most important contributor to hypotension, which should be assessed before CRRT initiation. It is recommended that the dosage of vasopressors should be adjusted appropriately to maintain blood pressure at least 10 mmHg above the target[36].

There were several limitations of this study. As a single-center study, the sample size of this study was relatively small, and the findings may not be widely applicable. Further validation with multicenter and large samples is needed. Besides, this study did not exclude patients with a history of hypotension during CRRT, which may have a potential impact on the results. This study was an observational study, susceptible to unmeasured biases and unknown confounding factors, a relationship between hypotension and the factors could not be inferred causally. The findings of this study are considered a primary reference for clinical practice.

Conclusion

This incidence of hypotension within the initial hour of CRR in critically ill patients was 38.2%. Multiple factors affect the occurrence of hypotension. Female, PLT, and baseline MAP are protective factors against hypotension, whereas age, mechanical ventilation, ultrafiltration rate of 101-200ml/h, INR, and myoglobin levels are risk factors for hypotension. More prospective studies are required to explore the factors necessary for the development of targeted therapies to prevent hypotension.

Acknowledgments

The authors would like to acknowledge the help provided by clinicians and nurses in the ICU during data collection, and the guidance of data analysis provided by Mengsheng Zhao from Nanjing Medical University.

References

1. XM C. Standard Operating Procedures for Blood Purification (2021 Edition). Peking: People's Medical Publishing House; 2021.

2. Ronco C, Reis T. Continuous renal replacement therapy and extended indications. Seminars in dialysis. 2021;34(6):550-60. doi: 10.1111/sdi.12963.

3. Bellomo R, Baldwin I, Ronco C, Kellum JA. ICU-Based Renal Replacement Therapy. Crit Care Med. 2021;49(3):406-18. doi: 10.1097/ccm.0000000000004831.

4. Sun LX HT, Gao Y, Dong Z. Effects of continuous renal replacement therapy on renal function, hemodynamics, and inflammatory factors in elderly patients with severe acute renal failure. Chinese Journal of Gerontology. 2019;39(24):6036-9.

5. Liu Z. LJ, Wu C., Yang J. The effect of continuous renal replacement therapy on hemodynamics and renal function in patients with severe acute renal failure. Chinese Journal of Clinical Physicians. 2016;44(02):50-2.

6. Li J. Study on risk factors and preventive nursing of hypotension during continuous renal replacement therapy in patients with acute kidney injury. General Nursing. 2022;20(13):1836-9. doi: 10.12104/j.issn.1674-4748.2022.13.029.

7. Reeves PB, Mc Causland FR. Mechanisms, Clinical Implications, and Treatment of Intradialytic Hypotension. Clinical journal of the American Society of Nephrology : CJASN. 2018;13(8):1297-303. doi: 10.2215/cjn.12141017.

8. MacEwen C, Sutherland S, Daly J, Pugh C, Tarassenko L. Relationship between Hypotension and Cerebral Ischemia during Hemodialysis. J Am Soc Nephrol. 2017;28(8):2511-20. doi: 10.1681/asn.2016060704.

9. Stefánsson BV, Brunelli SM, Cabrera C, Rosenbaum D, Anum E, Ramakrishnan K, et al. Intradialytic hypotension and risk of cardiovascular disease. Clinical journal of the American Society of Nephrology : CJASN. 2014;9(12):2124-32. doi: 10.2215/cjn.02680314.

10. Ling L ZZ, Qiu LH, Feng B. Influencing factors and the incidence of hypotension during continuous renal replacement therapy in critically ill patients. Journal of Nurses Training. 2021;36(11):1048-52. doi: 10.16821/j.cnki.hsjx.2021.11.019.

11. Nie A, Zhang S, Cai M, Yu L, Li J, Su X. Incidence and associated factors for hypotension during continuous renal replacement therapy in critically ill patients. Int J Nurs Pract. 2024:e13296. doi: 10.1111/ijn.13296.

12. Shawwa K, Kompotiatis P, Jentzer JC, Wiley BM, Williams AW, Dillon JJ, et al. Hypotension within one-hour from starting CRRT is associated with in-hospital mortality. J Crit Care. 2019;54:7-13. doi: 10.1016/j.jcrc.2019.07.004.

13. Kelly YP, Sharma S, Mothi SS, McCausland FR, Mendu ML, McMahon GM, et al. Hypocalcemia is associated with hypotension during CRRT: A secondary analysis of the Acute Renal Failure Trial Network Study. J Crit Care. 2021;65:261-7. doi: 10.1016/j.jcrc.2021.07.008.

14. Akhoundi A, Singh B, Vela M, Chaudhary S, Monaghan M, Wilson GA, et al. Incidence of Adverse Events during Continuous Renal Replacement Therapy. Blood purification. 2015;39(4):333-9. doi: 10.1159/000380903.

15. Chazot G, Bitker L, Mezidi M, Chebib N, Chabert P, Chauvelot L, et al. Prevalence and risk factors of hemodynamic instability associated with preload-dependence during continuous renal replacement therapy in a prospective observational cohort of critically ill patients. Ann Intensive Care. 2021;11(1):95. doi: 10.1186/s13613-021-00883-9.

16. Sharma S, Waikar SS. Intradialytic hypotension in acute kidney injury requiring renal replacement therapy. Seminars in dialysis. 2017;30(6):553-8. doi: 10.1111/sdi.12630.

17. Avolio AP, Butlin M, Walsh A. Arterial blood pressure measurement and pulse wave analysis—their role in enhancing cardiovascular assessment. Physiological measurement. 2010;31(1):R1. doi: 10.1088/0967-3334/31/1/R01.

18. Salmasi V, Maheshwari K, Yang D, Mascha EJ, Singh A, Sessler DI, et al. Relationship between Intraoperative Hypotension, Defined by Either Reduction from Baseline or Absolute Thresholds, and Acute Kidney and Myocardial Injury after Noncardiac Surgery: A Retrospective Cohort Analysis. Anesthesiology. 2017;126(1):47-65. doi: 10.1097/aln.0000000000001432.

19. Association CN. Nursing group standards for continuous renal replacement therapy. 2023.

20. Chen H WL, Han P, Zhang T, Zhou JX. Risk factors and predictive value of hypotension in patients with SA-AKI undergoing CRRT. Chinese Science and Technology Journal Database (Full-text Version), Medicine and Health. 2024;(1).

21. Chen M. Factors influencing the incidence of hypotension during continuous renal replacement therapy in critically ill patients. Chinese Science and Technology Journal Database (Full-text Version), Medicine and Health. 2022;(01):024-7.

22. Yildiz AB, Vehbi S, Covic A, Burlacu A, Covic A, Kanbay M. An update review on hemodynamic instability in renal replacement therapy patients. Int Urol Nephrol. 2023;55(4):929-42. doi: 10.1007/s11255-022-03389-w.

23. Kang MW, Kim S, Kim YC, Kim DK, Oh KH, Joo KW, et al. Machine learning model to predict hypotension after starting continuous renal replacement therapy. Scientific reports. 2021;11(1):17169. doi: 10.1038/s41598-021-96727-4.

24. Ramirez-Sandoval JC, Gaytan J, Arvizu-Hernandez M, Mejia-Vilet JM, Chavez PX, Correa-Rotter R, et al. Prolonged intermittent renal replacement therapy for AKI in COVID-19 patients with acute respiratory distress syndrome. Journal of the American Society of Nephrology. 2020;31:249-50.

25. Kompotiatis P, Shawwa K, Jentzer JC, Wiley BM, Kashani KB. Echocardiographic parameters and hemodynamic instability at the initiation of continuous kidney replacement therapy. J Nephrol. 2023;36(1):173-81. doi: 10.1007/s40620-022-01400-2.

26. Cui Y, Cheng H, Zhang S, Wan M, Xu Y. Value of some clinical parameters in predicting continuous renal replacement therapy-related hypotension. Zhonghua Wei Zhong Bing Ji Jiu Yi Xue. 2020;32(4):488-93. doi: 10.3760/cma.j.cn121430-20200123-00085.

27. Shantsila E, Lip GYH, Shantsila A, Kurpas D, Beevers G, Gill PS, et al. Antihypertensive treatment in people of very old age with frailty: time for a paradigm shift? Journal of Hypertension. 2023;41(10):1502-10. doi: 10.1097/hjh.0000000000003495.

28. Medina-Liabres KRP, Kim S. Continuous renal replacement therapy in elderly with acute kidney injury. The Korean journal of internal medicine. 2020;35(2):284-94. doi: 10.3904/kjim.2019.431.

29. Jozwiak M, Teboul JL. Heart-Lungs interactions: the basics and clinical implications. Ann Intensive Care. 2024;14(1):122. doi: 10.1186/s13613-024-01356-5.

30. Woodcock TE, Woodcock TM. Revised Starling equation and the glycocalyx model of transvascular fluid exchange: an improved paradigm for prescribing intravenous fluid therapy. British journal of anaesthesia. 2012;108(3):384-94. doi: 10.1093/bja/aer515.

31. Li S, Lu Z, Wu S, Chu T, Li B, Qi F, et al. The dynamic role of platelets in cancer progression and their therapeutic implications. Nat Rev Cancer. 2024;24(1):72-87. doi: 10.1038/s41568-023-00639-6.

32. Vincent JL, Sakr Y, Singer M, Martin-Loeches I, Machado FR, Marshall JC, et al. Prevalence and

Outcomes of Infection Among Patients in Intensive Care Units in 2017. Jama. 2020;323(15):1478-87. doi: 10.1001/jama.2020.2717.

33. Group CSoNE. Expert consensus on volume assessment and management of continuous renal replacement therapy. Chinese Journal of Nephrology 2024;40(01):74-81.

34. Cecconi M, De Backer D, Antonelli M, Beale R, Bakker J, Hofer C, et al. Consensus on circulatory shock and hemodynamic monitoring. Task force of the European Society of Intensive Care Medicine. Intensive Care Med. 2014;40(12):1795-815. doi: 10.1007/s00134-014-3525-z.

35. Douvris A, Zeid K, Hiremath S, Bagshaw SM, Wald R, Beaubien-Souligny W, et al. Mechanisms for hemodynamic instability related to renal replacement therapy: a narrative review. Intensive Care Med. 2019;45(10):1333-46. doi: 10.1007/s00134-019-05707-w.

36. Zhou X, Wang Q, He Z, Xiao S. Impact of Double-Machine Replacement Protocol at Start of Continuous Renal Replacement Therapy in Vasopressor-Dependent Patients: A Retrospective Cohort Study. Blood purification. 2022;51(11):959-66. doi: 10.1159/000522258.

Supporting information

S1 Table. Comparison between IDH and non-IDH group

S2 Table. The coefficients of LASSO Regression Analysis

Figure Click here to access/download;Figure;FIGURES.docx

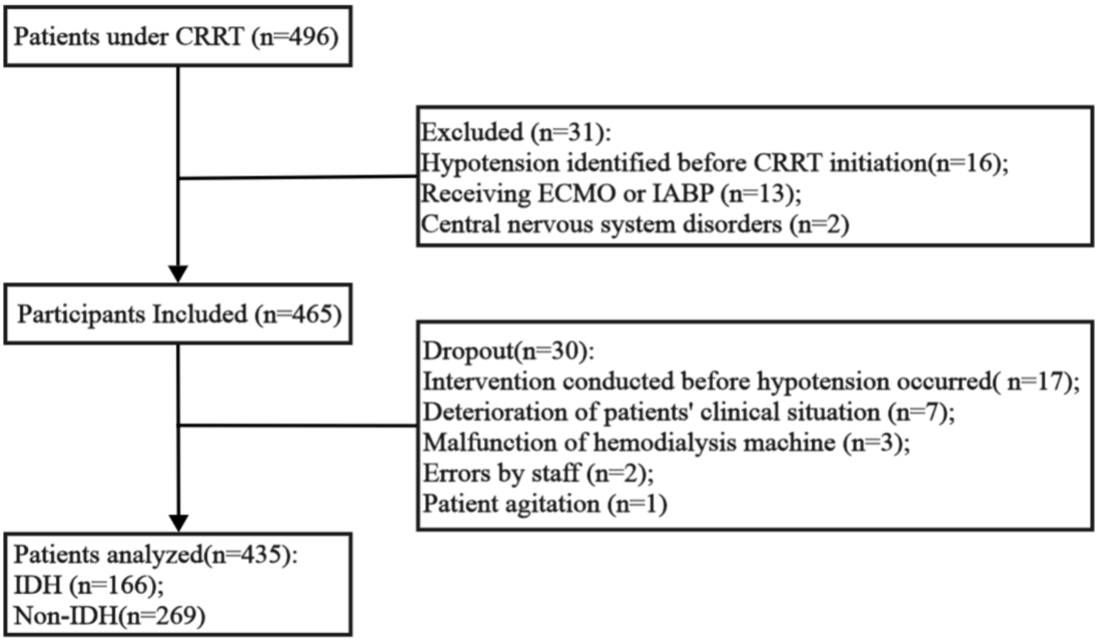

Figure 1 Flow Chart of Participant Inclusion and Exclusion

*CRRT, continuous renal replacement therapy; ECMO, extracorporeal membrane oxygenation; IABP, intra-aortic balloon pump

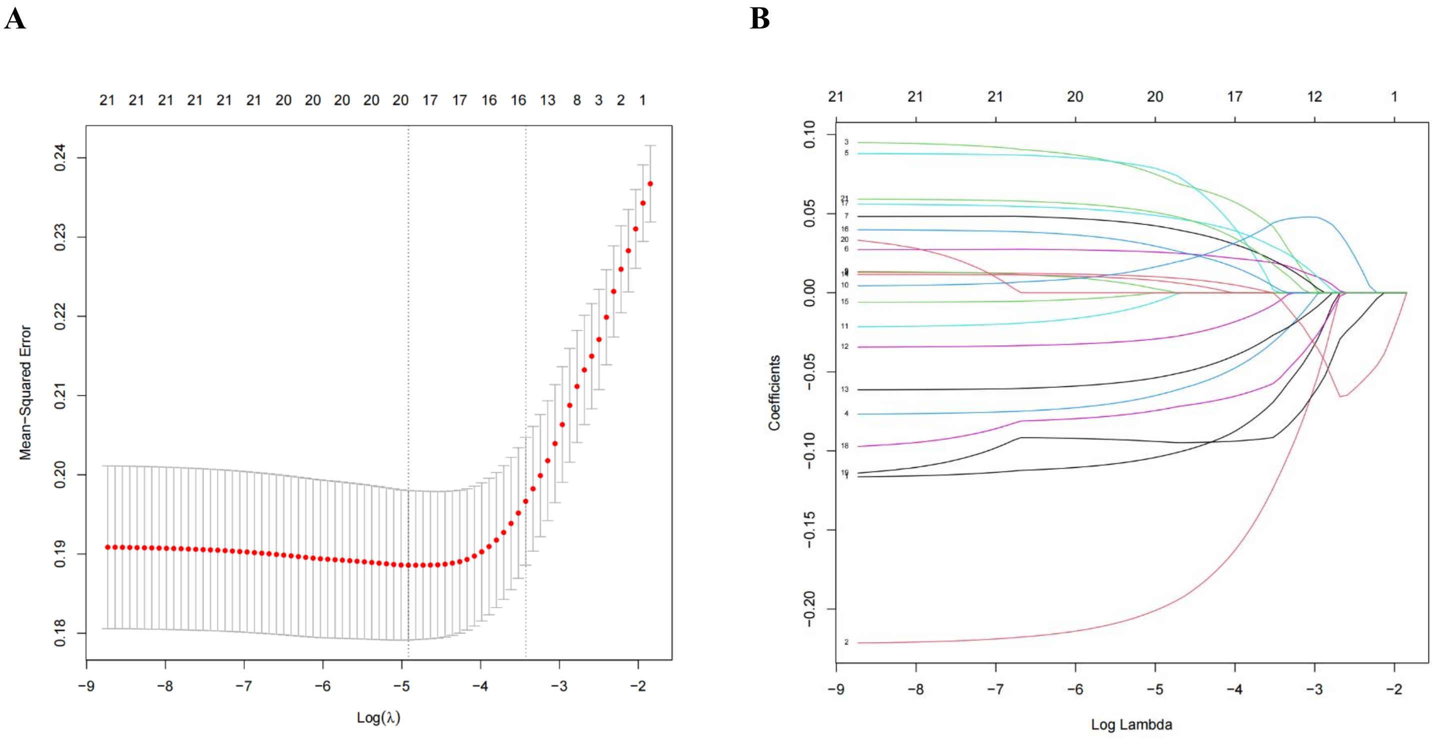

FIGURE 2 Results of The LASSO Regression Analysis

*A: Tunning Parameter (λ) Selection Cross-validation Error Curve; B: LASSO Regression Coefficient Path Plot

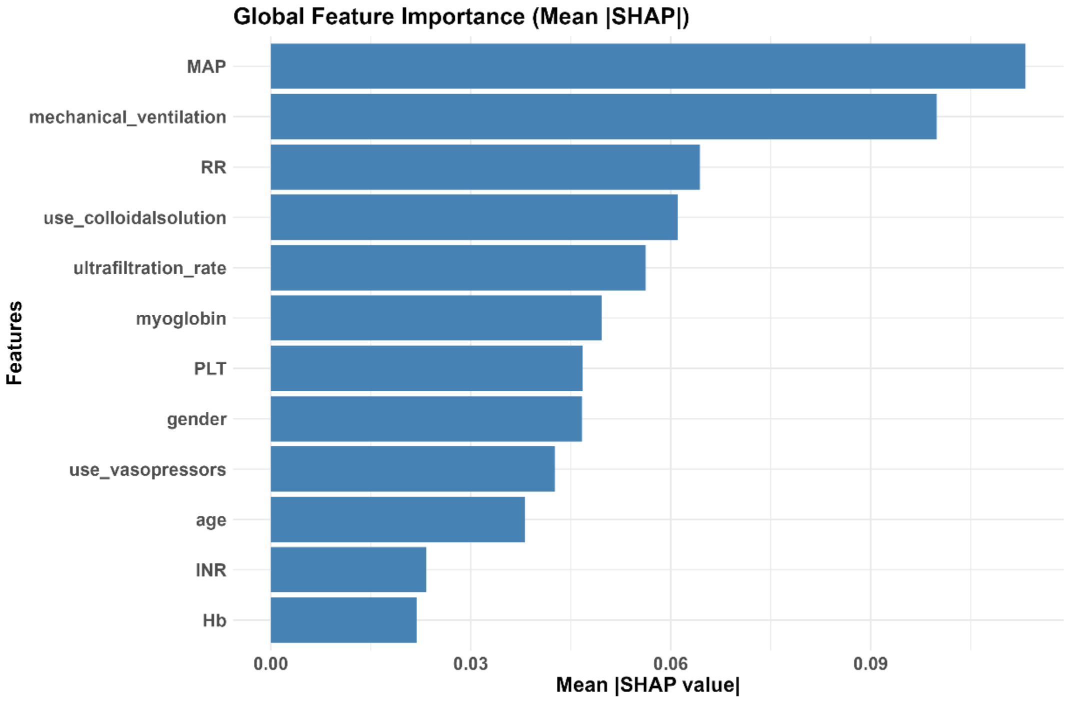

Figure 3 Importance of Predictors Based on SHAP Value

*MAP, mean arterial pressure; RR, respiratory rate; PLT, platelet count; INR, international normalized ratio; Hb, hemoglobin.

Click here to access/download
Supporting Information
supporting information.docx
